# Single-cell RNA sequencing in diabetic foot ulcers: pathogenesis, biomarkers, and translational potential

**DOI:** 10.3389/fcdhc.2026.1833678

**Published:** 2026-09-03

**Authors:** Shuhan Yang, Siqian Wang, Ruiyi Yang, Ke Xu, Xiaofeng Yang, Yifeng Zhao

**Affiliations:** 1 Emergency Department of the Second Affiliated Hospital of Chongqing Medical University, Chongqing, China; 2 Chongqing Nursing Vocational College, Chongqing, China; 3 Department of Gastroenterology, The First Affiliated Hospital of Chongqing Medical University, Chongqing, China

**Keywords:** diabetic foot ulcer, extracellular matrix remodeling, macrophage polarization, single-cell sequencing, wound healing

## Abstract

Diabetic foot ulcer (DFU), a common diabetic complication driven by impaired angiogenesis, neuropathy and metabolic disorders, has complex pathogenesis and poses great challenges to clinical management. Single-cell RNA sequencing (scRNA-seq), an emerging transcriptomic technique, allows disease profiling at single-cell resolution. This review summarizes recent advances in scRNA-seq-based DFU research, revealing that JMJD3-mediated macrophage polarization failure and dysregulated IGF-1-SP1-CD248 pathway hinder wound repair, with *MMP1, MMP3, MMP11*, JMJD3 and *HIF1A* identified as valuable biomarkers and therapeutic targets. It highlights scRNA-seq’s utility in unraveling pathogenic mechanisms, discovering therapeutic targets and biomarkers, and facilitating precision medicine, to provide new insights and research directions for DFU therapy.

## 1 Introduction: diabetic foot

Diabetes is one of the fastest-growing chronic non-communicable diseases worldwide, with a rapidly increasing number of affected individuals. According to the 2021 International Diabetes Federation report, approximately 537 million adults aged 20–79 years have diabetes, corresponding to a global prevalence of 10.5%. This number is projected to rise to 643 million by 2030 and 783 million by 2045. The growth is especially pronounced in low- and middle-income countries, with China, India, and Pakistan reporting the highest case numbers (1). China alone has more than 118 million people with diabetes, representing 22% of the global burden (2). Diabetes causes approximately 6.7 million deaths annually and leads to complications in about 40% of patients (1). Among these complications, diabetic foot is one of the most severe and represents a major challenge to global public health systems. Diabetic foot refers to foot lesions occurring in patients with current or previous diabetes (3). Globally, approximately 15% of patients with diabetes are at high risk of developing diabetic foot, and 5% experience diabetic foot pain. Diabetic foot ulcer (DFU) is the most common clinical manifestation of diabetic foot. In China, the prevalence of diabetic foot among patients with diabetes is 8.1%, with over one million new ulcer cases annually and an amputation rate of 21.3%, markedly higher than that reported in Europe and the United States (4–6). Diabetic foot is frequently complicated by infections, with approximately 60% of ulcers becoming infected (7). Among patients with mild to moderate infections, approximately 20% develop osteomyelitis, whereas 50–60% of severe cases do so (8), which triples the risk of systemic inflammatory response and sepsis (9). In addition to high amputation and infection rates, diabetic foot is associated with substantial mortality. Studies indicate that the 5-year mortality among patients with diabetic foot approaches 50% (10), while the 3-year survival after amputation is below 50% (11). Among patients with DFUs, 15–25% eventually require amputation (4, 12). Further research on DFUs is therefore urgently needed to reduce the global disease burden and improve healing outcomes.

The disease spectrum of diabetic foot includes peripheral neuropathy, peripheral arterial disease, foot infection, foot ulcer, neuro-osteoarthropathy, foot gangrene, and, in severe cases, lower-limb amputation. DFU results from complex interactions among neuropathy, peripheral arterial disease, and impaired wound healing; it is often accompanied by peripheral neuropathy and/or lower-limb peripheral arterial disease (13). Vascular dysfunction and immune dysregulation are major contributors to this poor prognosis (10). Type 2 diabetes, advanced age, smoking, poor glycemic control, insulin use, longer diabetes duration, and elevated glycated hemoglobin levels (14–16) all increase the risk of DFU. Patients with type 2 diabetes, males, and non-white ethnic groups, including Black and Hispanic populations, have a higher incidence risk (16, 17). Socioeconomic and geographic disparities also indirectly influence disease occurrence (18). Cardiovascular and kidney diseases not only increase the risk of DFU but also delay wound healing, increase ulcer recurrence, and raise the likelihood of lower-limb amputation (4, 12).

**Figure 1:**
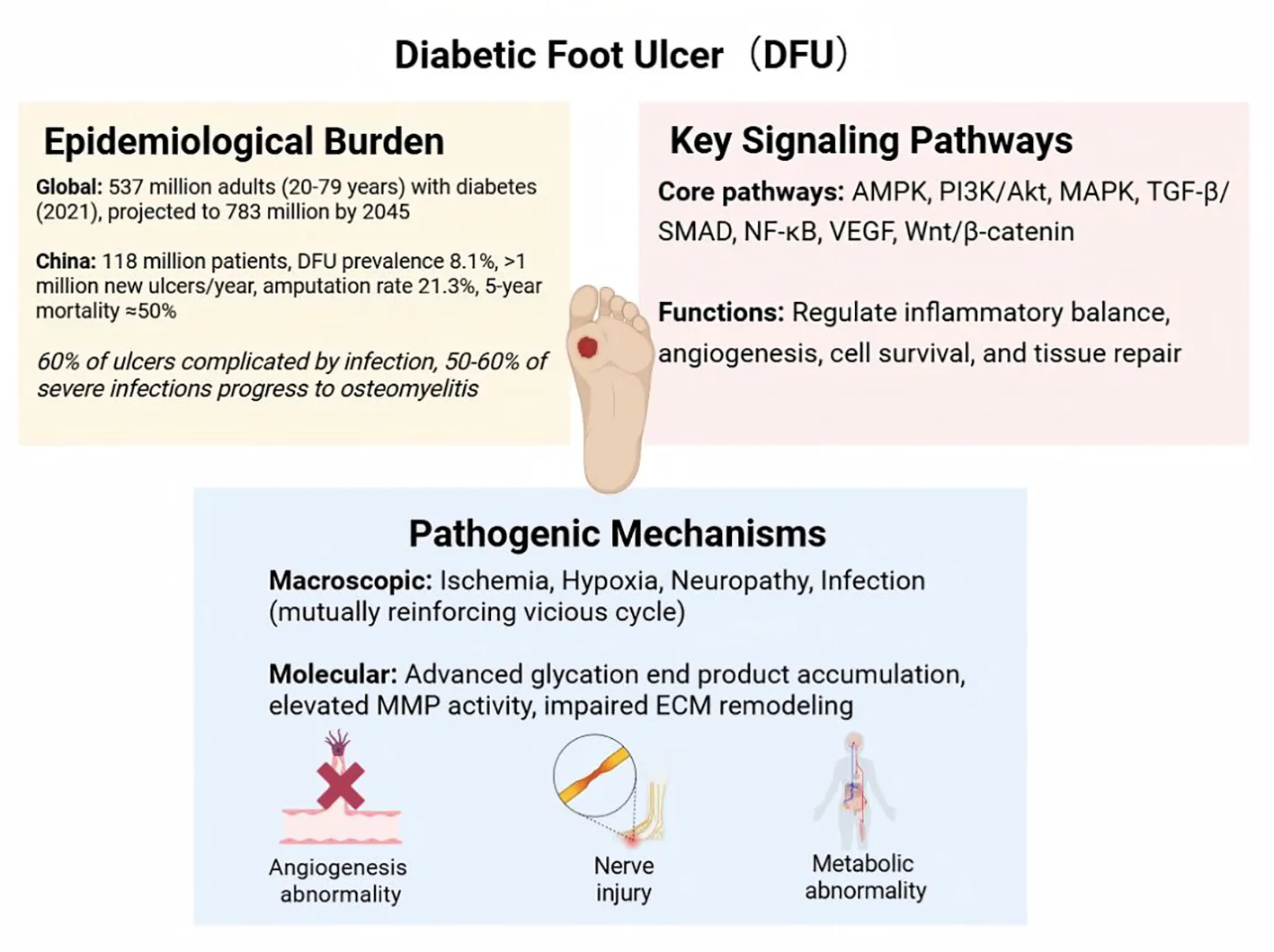
Schematic summary of diabetic foot ulcer (DFU). The yellow region presents global and Chinese epidemiological data of diabetes and DFU incidence, infection, amputation and mortality risks. The blue region illustrates the mutual vicious cycle of ischemia, hypoxia, neuropathy and infection, as well as molecular abnormalities including AGE accumulation and disordered ECM remodeling. The pink box lists core signaling pathways that jointly control inflammatory balance, angiogenesis and skin repair. Created with BioRender.com.

Existing diagnostic and therapeutic methods for diabetic foot ulcers are mostly based on macroscopic tissue analysis, making it difficult to accurately identify the heterogeneity of cell subpopulations and thoroughly elucidate the pathological evolution of the disease and the regulatory mechanisms of cell interactions, which restricts the analysis of pathogenesis and the development of targeted diagnostic and therapeutic schemes. Single-cell sequencing can focus analysis at the single-cell scale, and with its ultra-high resolution, it can accurately reconstruct the entire pathological development process of diabetic foot ulcers and systematically analyze the interaction patterns between cells, becoming a valuable emerging approach for mechanism exploration in this field. Currently, single-cell sequencing technology is developing rapidly, and its application achievements in basic research and clinical exploration of diabetic foot ulcers are constantly emerging. How to systematically summarize the current status of translational research related to this technology and leverage its technological advantages to overcome clinical challenges such as wound healing difficulties has become an important issue that needs urgent exploration. This paper does not cover other diabetic complications or non-single-cell omics studies. It aims to provide a cellular-level reference for mechanistic research and clinical translation in DFU (**Figure 1**).

### 1.1 Pathogenesis

DFU pathogenesis is mediated by multiple interacting factors, with peripheral neuropathy, limb ischemia and hypoxia, and recurrent local infection serving as the core systemic and tissue-level pathological basis (**Figure 1**). These conditions aggravate metabolic disturbances, nutrient-transport imbalance, excessive accumulation of advanced glycation end products (AGEs), and increase matrix metalloproteinase (MMP) activity, thereby creating a chronic wound microenvironment characterized by impaired healing and delayed resolution. Collectively, these changes disrupt extracellular matrix (ECM) remodeling and normal skin tissue repair. Diabetic peripheral neuropathy manifests as sensory, motor, and autonomic dysfunction, all of which contribute to ulcer formation and progression to chronicity (19). Sensory neuropathy leads to the loss of protective sensations such as pain, touch, and temperature perception in the feet, resulting in unnoticed minor injuries such as trauma, abrasions, pressure, and burns. These hidden injuries persist, ultimately promoting ulcer development (20). Motor neuropathy causes abnormal musculoskeletal changes, including denervation of intrinsic foot muscles, reduced muscle strength, and atrophy. It also leads to foot deformities such as hammertoes, claw toes, and Charcot foot. These deformities disrupt normal plantar-pressure distribution and, when combined with loss of protective sensation, render patients unable to perceive excessive pressure or injury, thereby increasing susceptibility to skin breakdown and DFU progression (21). Autonomic dysfunction reduces sweating in the foot skin, causing dryness, fissuring of the stratum corneum, decreased skin elasticity, and impaired skin integrity, which facilitate bacterial entry. Additionally, arteriovenous shunting impairs blood-flow regulation, further affecting tissue perfusion and wound healing.

The ischemic and hypoxic changes in diabetic foot ulcers primarily stem from systemic and lower-extremity vascular lesions induced by diabetes. Prolonged hyperglycemia triggers atherosclerosis in large lower limb vessels, leading to arterial luminal stenosis and impaired blood flow delivery, which significantly reduces overall blood perfusion in the feet. Concurrently, diabetes also affects systemic microvasculature, disrupting distal microcirculatory pathways and further diminishing blood supply reserves in the foot tissues (19). Insufficient arterial blood supply to the lower limbs, coupled with microcirculatory perfusion impairment, results in inadequate oxygenation and slowed nutrient delivery to foot tissues, while metabolic waste from tissues cannot be promptly eliminated. Under this persistent state of ischemia and hypoxia, the skin and soft tissues of the feet exhibit markedly reduced tolerance to damage, making them prone to tissue breakdown from minor trauma or localized pressure. The ischemic-hypoxic condition disrupts normal wound repair processes, and insufficient local blood flow reserves fail to sustain the circulatory compensation required for wound healing, ultimately leading to ulcer formation and persistent non-healing.

In addition, diabetic foot ulcers are prone to recurrent infection. Long-term hyperglycemia can cause lower-limb blood-circulation disorders, insufficient local tissue perfusion, decreased tissue oxygen supply and nutrient metabolism, damage to the skin barrier structure and defense function of the feet, and significantly reduce the ability to resist external pathogenic microorganisms (22). Under the condition of diabetes, the overall immune defense function of the body is abnormal, and the inflammatory regulation mechanism is disordered, which cannot effectively eliminate invasive pathogens. Abnormally elevated inflammatory mediators in the wound area continuously amplify the local inflammatory response, aggravate tissue inflammatory damage, and further impair the wound repair microenvironment. Bacteria are prone to colonize and proliferate in damaged wounds, local infections; infection in turn aggravates tissue blood-circulation disorder and necrosis, forming a vicious pathological cycle of infection, tissue destruction, and blocked healing, which will eventually lead to recurrent infection of diabetic foot ulcer and prolonged wound healing (23).

On the basis of macroscopic tissue lesions, DFU wounds exhibit a series of cellular functional abnormalities and molecular signal regulation disorders. First, the hypoxia response mechanism of diabetes wound is impaired, which is manifested by the down-regulation of hypoxia-inducible factors (HIF-1, HIF-1α), abnormal accumulation of phenylpyruvate, and the activation of macrophages through the CD36–PPT1–NLRP3 signaling axis, inducing a persistent inflammatory response. Meanwhile, excessive MMP activation accompanied by insufficient collagen synthesis directly hinders extracellular matrix remodeling. The transcription factor Nrf2 is a core regulatory molecule in oxidative stress and the inflammatory response. Under physiological conditions, Nrf2 is retained in the cytoplasm by Keap1 and degraded by proteasomes; when oxidative stress occurs, Keap1 and Nrf2 dissociate, Nrf2 enters the nucleus to start the expression of antioxidant enzymes and promote the synthesis of glutathione, and Nrf2 mutation significantly increases the risk of diabetic neuropathy, kidney disease, foot ulcer and microvascular complications (24). Non-coding RNAs are also involved in the progression of DFU, such as downregulation of miR-146a expression, which can exacerbate local inflammation, oxidative stress, and endoplasmic reticulum stress by regulating PI3K/Akt; Sirtuin-6 (SIRT6) can accelerate wound healing by activating the Nrf2 pathway, while abnormal upregulation of PLIN1 can increase inflammatory markers and drive local inflammatory responses through the NF-κB pathway (24–28).

Angiogenesis is a key cellular process determining whether DFU wounds can heal. Vascular endothelial growth factor (VEGF) promotes neovascularization, whereas FGF-1, FGF-2, TGF-β1, angiopoietin-2, and ECM components synergistically regulate endothelial cell proliferation and vessel formation. Dysregulation of PI3K/Akt, MAPK, TGF-β/Smad, Notch, NF-κB, VEGF, Wnt/β-catenin, and Nrf2 signaling can inhibit cell survival, delay inflammation resolution, and impair angiogenesis. During the middle and late stages of wound repair, angiogenesis normally subsides as granulation tissue transforms into collagen-rich scar tissue; however, aberrantly high expression of anti-angiogenic factors, including thrombospondin-1 (TSP1), Sprouty2, and pigment epithelium-derived factor (PEDF), further suppresses angiogenesis. Dysregulated microRNA expression disturbs the balance between pro- and anti-angiogenic factors, whereas stem-cell-derived exosomes may improve healing by enhancing collagen deposition, reducing inflammation, and promoting vascular regeneration. Specific endothelial cell clusters expressing SLCO2A1 and CYP1A1 have also been proposed as potential therapeutic targets in DFU (29). Fibroblasts, as core effector cells in wound repair, are highly heterogeneous and plastic and regulate inflammation, angiogenesis, and ECM deposition. Resident and blood-derived fibroblasts migrate to the wound in response to chemotactic signals and participate in tissue repair through proliferation and differentiation. α-SMA^+^ myofibroblasts mediate wound contraction and collagen synthesis, while interactions with endothelial cells and keratinocytes support granulation tissue formation and skin regeneration. Fibroblast growth factors further amplify these repair processes (30).

At present, several innovative therapeutic strategies have emerged for DFU, including growth factor intervention, nanomedicine, microRNA-targeted therapy, novel wound dressings, stem cell transplantation, and immunomodulation, which target ischemia, hypoxia, chronic inflammation, and molecular dysregulation. These approaches have shown promising application prospects in both basic experiments and clinical research (31). However, the fine molecular regulatory networks driving DFU healing remain incompletely characterized. Further cellular-level studies are needed to identify novel targets and develop cost-effective interventions that improve wound-healing rates in patients with DFU.

### 1.2 Current diagnosis and treatment status

The current diagnostic approaches for DFUs encompass neurological examinations, pressure tests, imaging modalities, and transcutaneous oxygen pressure measurements. These assessments are first-line tools for screening and routine evaluation of diabetic peripheral neuropathy and diabetic foot and are widely used in endocrinology departments and specialized foot centers (32). Initial evaluation includes inspection of the skin, as well as assessment of ulcer depth and extent, to determine severity. Vascular function is typically assessed using the Ankle-Brachial Index and transcutaneous oxygen pressure, while nerve conduction velocity tests are employed to evaluate the degree of neuropathy (33). The above has been included as a routine clinical practice. Imaging techniques such as CT and MRI are first-line clinical imaging methods widely used to screen for deep soft tissue infections, abscesses, and osteomyelitis in the feet. They have been widely applied in tertiary hospitals at all levels. More recently, contrast-enhanced ultrasound imaging has only been tested in some large tertiary foot disease centers for the precise evaluation of microcirculation damage in the feet of DFU patients, and has not yet been fully adopted in primary hospitals.

In the standard treatment strategy, strict control of metabolic indicators such as blood glucose, blood pressure, and blood lipids, combined with local wound management (32), is a globally recognized basic clinical approach. Surgical debridement and regular dressing changes are routine basic clinical procedures that create favorable conditions for wound healing by removing necrotic tissue and wound exudate (33).

Antibiotic therapy is tailored to infection status, and agents that improve microcirculation can enhance vascular dilation, blood flow, and cellular migration at the ulcer site. It is currently the mainstream conservative treatment plan in clinical practice. For severe lower limb arterial occlusive disease, endovascular intervention therapy and lower limb bypass grafting have become mature clinical procedures in vascular surgery; when the condition progresses to advanced irreversible gangrene, amputation surgery is an important clinical intervention to save the patient’s life. In addition, mesenchymal stem cell therapy has attracted increasing attention. For instance, Han et al. demonstrated that mesenchymal stem cell–derived exosomes carrying Krüppel-like factor 3 antisense RNA1 promote wound healing by enhancing angiogenesis (34). This approach has not yet been incorporated into routine clinical treatment and cannot be widely applied in clinical practice.

Compared with ordinary wounds, the treatment of DFUs is considerably more challenging due to the complexity of their impaired healing mechanisms, which results in delayed recovery (32). Current research has primarily focused on dysregulation of inflammation, angiogenesis, and cellular function. Against this background, single-cell sequencing technology offers new opportunities to elucidate the underlying mechanisms, identify therapeutic targets, and guide the development of innovative treatments and early diagnostic strategies.

## 2 Single-cell sequencing

### 2.1 The advantages of single-cell sequencing in disease research

**Figure 2:**
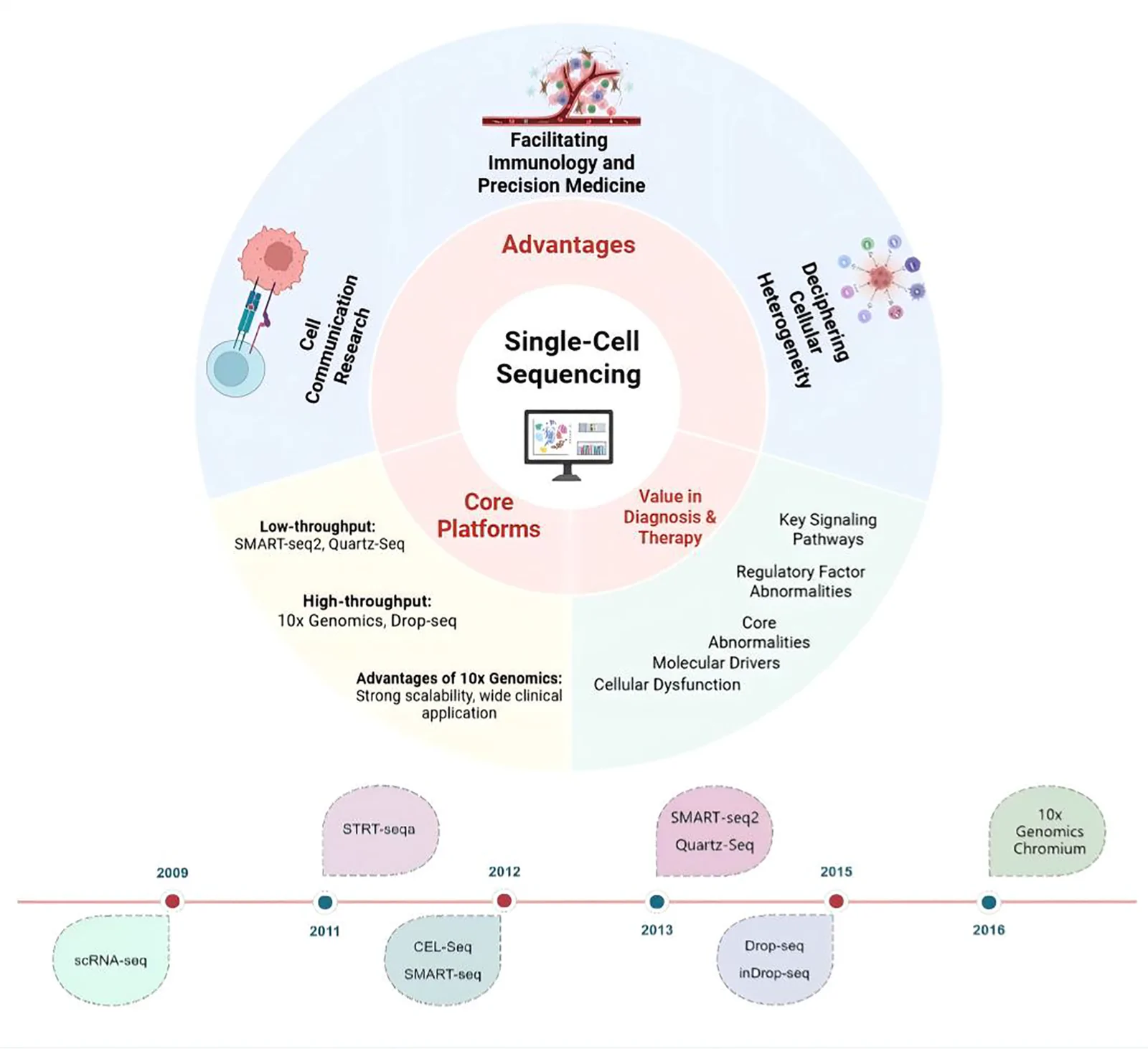
Integrative schematic of single-cell RNA sequencing (scRNA-seq). The central part shows the core concept of scRNA-seq; surrounding panels describe its core strengths: dissecting cell heterogeneity, analyzing intercellular communication, advancing immunology and precision medicine, and mining therapeutic targets. Two types of sequencing platforms (low-throughput and high-throughput 10x Genomics) are classified, and the bottom timeline records key technological innovations from 2009 to 2016. Created with BioRender.com.

ScRNA-seq has emerged as a powerful tool for dissecting transcriptional heterogeneity within complex tissues. By profiling gene expression at single-cell resolution, scRNA-seq enables the identification of distinct cellular subpopulations and reveals specific interactions between different cell types across a wide range of diseases, including neurodegenerative disorders, cardiovascular diseases, and cancers (35). Beyond defining cell states, scRNA-seq provides insights into the immune microenvironment and clinical pathology, offering valuable clues for the development of novel immunotherapies through the analysis of intercellular communication (36). Moreover, scRNA-seq data can be integrated into the construction of prognostic models and risk scores, thereby advancing precision medicine and patient stratification. Mapping of single-cell atlases under both physiological and pathological conditions further uncovers unique cellular ecosystems, deepening our understanding of disease pathogenesis at the cellular level (37) (**Figure 2**).

The growing availability of scRNA-seq and single-nucleus RNA sequencing (snRNA-seq) datasets has driven the development of computational tools to analyze intercellular communication. Platforms such as CellChat, iCELLNET, iTALK, and SingleCellSignalR allow the systematic exploration of ligand–receptor interactions and signaling network alterations, providing critical insights into regional heterogeneity and disease formation (35). In parallel, integration with other high-throughput omics approaches—including proteomics, bulk RNA-seq, and spatial transcriptomics in both preclinical and clinical contexts—has provided mechanistic insights into tissue remodeling and the efficacy of emerging therapies, such as novel wound dressings (38).

In previous DFU studies, conventional bulk transcriptomic and proteomic approaches have reflected overall molecular changes in wound tissue but have inherent limitations in the highly heterogeneous DFU microenvironment. Bulk sequencing measures average tissue-level transcription, thereby masking cellular-subpopulation heterogeneity and preventing attribution of differential signals to specific cellular sources (38). Bulk RNA sequencing rapidly acquires global transcriptional profiles of tissues to provide a panoramic baseline reference. ScRNA-seq resolves cellular heterogeneity and uncovers transcriptional differences among diverse cell subsets within mixed tissues. Spatial transcriptomics maps cell clusters obtained from dissociated sequencing back to the native tissue architecture, while proteomics validates transcriptomic predictions at the protein level. Integrative multi-omics enables multi-dimensional cross-verification of molecular changes, avoids bias from individual techniques, and comprehensively illustrates the mechanisms underlying impaired wound repair in DFU. Proteomic analyses are limited by the sensitivity of low-abundance protein detection, which makes it difficult to capture rare immune subsets or precisely localize cell types (39). DFU wounds consist of complex networks of immune, stromal, endothelial, and other cell types. Traditional approaches cannot adequately identify functional differentiation of immune subsets such as T cells and macrophages, rare cell populations, or intercellular communication patterns (40). scRNA-seq overcomes these limitations by enabling unbiased identification of cellular subpopulations at single-cell resolution and precise characterization of cellular heterogeneity, transcriptional states, and regulatory networks in DFU. Rare subpopulations, cell-specific pathways, and spatial-distribution information revealed by scRNA-seq are core data that cannot be obtained through, or substituted by, conventional bulk approaches, providing essential technical support for elucidating immune imbalance and repair barriers in DFU (38, 41).

In the context of DFUs, scRNA-seq offers unique advantages by capturing the transcriptional dynamics of individual cells within the wound microenvironment. This approach allows the identification of cell subtypes, signaling pathways, and regulatory networks involved in impaired wound healing. By characterizing the roles of fibroblasts, keratinocytes, immune cells, and endothelial populations at single-cell resolution, scRNA-seq helps elucidate how their interactions influence inflammation, angiogenesis, and tissue repair. Furthermore, the identification of gene signatures and immune-regulatory mechanisms provides candidate therapeutic targets, offering new opportunities for personalized interventions in DFU management (42).

### 2.2 The value of single-cell sequencing in diagnostic and therapeutic research

Single-cell sequencing has played a central role in advancing oncology by enabling a deeper understanding of tumor biology and immune responses at the cellular level. For instance, Zheng et al. applied scRNA-seq to T cells from hepatocellular carcinoma patients, uncovering key gene signatures of exhausted CD8^+^ T cells and tumor-specific regulatory T cells (Tregs), which have provided valuable insights into tumor-infiltrating lymphocytes and guided the development of targeted immunotherapies (43). Similarly, scRNA-seq has revealed the heterogeneity of Tregs in non-small cell lung cancer, highlighting functional diversity and helping refine immune regulation strategies in cancer (44). Moreover, scRNA-seq has identified macrophages and dendritic cells as key mediators of cellular interactions in colorectal cancer, driving the development of myeloid-targeted therapies (45).

Building on these advancements in oncology, single-cell sequencing is now making significant contributions to the study of DFUs. Much like its role in cancer, scRNA-seq is providing a detailed map of immune cell populations, fibroblasts, and endothelial cells in DFU tissues. It has revealed key alterations in immune cell activation, chronic inflammation, and impaired wound healing, similar to the immune dysregulation seen in tumors. By offering a high-resolution view of the cellular landscape in DFUs, scRNA-seq is helping identify new therapeutic targets to promote healing and tissue regeneration, much like its role in advancing cancer therapies. These findings underscore the power of single-cell sequencing to enhance our understanding and treatment of complex diseases, from cancer to chronic wounds such as DFU (46).

From a clinical application perspective, scRNA-seq remains primarily at the stage of basic research and clinical translational investigation and has not yet been incorporated into standardized systems for routine tumor diagnosis or efficacy evaluation. High testing costs, lack of unified experimental standards, complex data-analysis pipelines, and insufficient prospective validation in large clinical cohorts currently prevent scRNA-seq from routine clinical use. However, novel molecular markers and immune targets discovered by this technology, such as *LAYN*, *TNFRSF9*, *LAG-3*, and *TIGIT*, have gradually entered clinical trials of anticancer immunotherapy. In addition, tumor-infiltrating lymphocyte (TIL) selection and CAR-T manufacturing strategies screened or optimized using single-cell sequencing have been explored in advanced solid tumors. Thus, related derivative technologies and targeted therapies are gradually transitioning from basic research toward clinical application (47–49) (**Figure 2**).

## 3 Application of single-cell sequencing in diabetic foot ulcers

**Figure 3:**
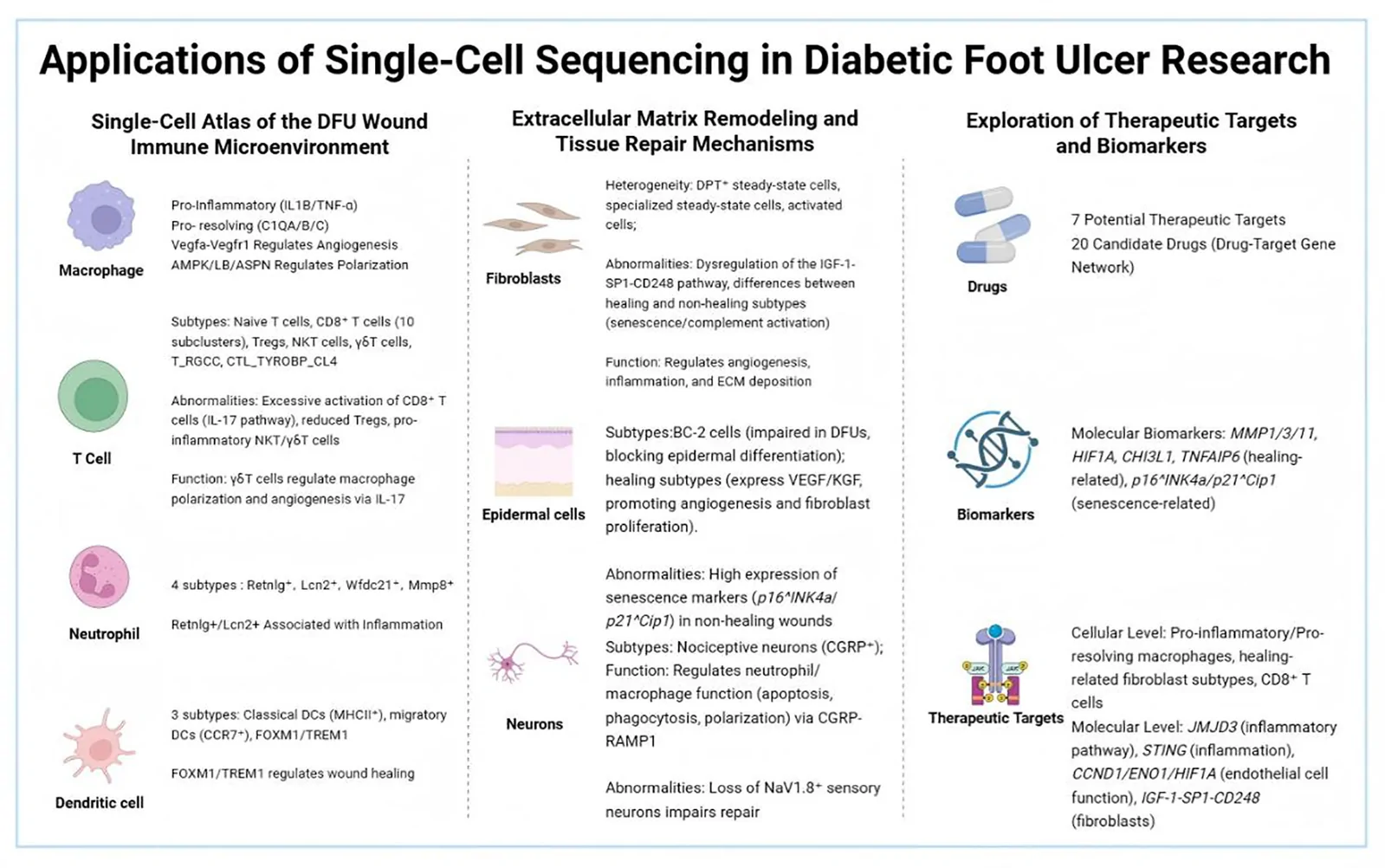
Three core research directions of scRNA-seq in DFU research. Left panel shows the subtypes, functional features and regulatory signals of wound immune cells (macrophages, T cells, neutrophils, dendritic cells). Middle panel depicts ECM remodeling and repair functions of fibroblasts, epidermal cells and sensory neurons. Right panel displays candidate therapeutic targets, small-molecule drugs and multi-layer biomarkers identified via single-cell transcriptome analysis. Created with BioRender.com.

A comprehensive literature search was conducted in PubMed, Web of Science Core Collection, and Embase from database inception to December 2025. The search combined Medical Subject Headings (MeSH) and free-text terms, including diabetic foot ulcer (DFU), single-cell RNA sequencing (scRNA-seq), immune microenvironment, wound healing, and biomarkers. Original research articles and high-quality reviews focusing on scRNA-seq in DFU were included if they involved human clinical samples or diabetic animal models and addressed pathogenesis, cellular heterogeneity, signaling pathways, or biomarkers. Conference abstracts, case reports, duplicate publications, articles without accessible full text, and studies irrelevant to the topic were excluded. Eligible studies were synthesized after duplicate removal, title/abstract screening, and full-text review (**Figure 3**).

### 3.1 Single-cell atlas of the wound immune microenvironment

In the context of DFUs, single-cell sequencing has been particularly valuable in characterizing the composition and spatial distribution of diverse cellular populations within the pathological wound environment. Persistent hyperglycemia imposes systemic metabolic stress, which drives chronic inflammation persisting throughout disease progression. As a result, immune-related cell populations have become a primary focus of single-cell transcriptomic studies. The construction of high-resolution single-cell atlases provides a systematic framework for delineating the wound immune microenvironment, offering rigorous insights into the cellular and molecular mechanisms underlying impaired healing in DFUs (**Figure 3**).

#### 3.1.1 Macrophages

Macrophages are central regulators of tissue repair during skin inflammation, yet their high plasticity is compromised under pathological conditions such as diabetes, resulting in a sustained pro-inflammatory state (50). In diabetic patients, hyperglycemia, ischemia, hypoxia, and microenvironmental alterations result in increased macrophage infiltration and aberrant phenotypes that can both promote and inhibit inflammation. Overall, relevant studies show a consistent trend: macrophages are broadly classified into two functional states: classically activated pro-inflammatory macrophages and alternatively activated pro-resolving macrophages. Pro-inflammatory macrophages dominate in the early stages of wound healing and exert pro-inflammatory and antimicrobial effects through oxidative stress and cytokine release (51), including TNF-α and IL-1β. Their activity is regulated by inflammatory mediators such as *IL1B, S100A8, and S100A9*. Epigenetic modulation also contributes to their function; for example, the histone demethylase JMJD3 enhances inflammatory gene expression via the JAK1/3–STAT3 pathway (52) (STZ-induced type 1 and db/db type 2 diabetic mice), with IL-6 amplifying this effect and promoting NF-κB–mediated transcription through H3K27me3 demethylation. Inhibition of JMJD3 in macrophages alleviates inflammation and improves diabetic foot ulcer wound repair in both streptozotocin (STZ)-induced type 1 and db/db type 2 diabetic mouse models (53–55).

By contrast, pro-resolving macrophages secrete anti-inflammatory mediators, including *C1QA/B/C* and *CD163*, thereby suppressing inflammation and facilitating tissue remodeling (56). During DFU progression, both pro-inflammatory and pro-resolving macrophage subpopulations increase. Impaired transition from pro-inflammatory to pro-resolving polarization is a core cellular mechanism underlying persistent chronic inflammation and delayed repair, a conclusion supported by multiple *in vivo* animal studies (STZ, db/db mice) and human clinical scRNA-seq datasets (57). Beyond their intrinsic functions, macrophages actively interact with other immune and stromal cells. They secrete chemokines that recruit neutrophils and coordinate inflammatory intensity and duration (58), while also supporting endothelial cell-mediated anti-inflammatory niches (59). Pro-resolving macrophages promote angiogenesis by remodeling the ECM, recruiting vascular-associated cells, and engaging in ligand–receptor interactions such as VEGFA–VEGFR1, as revealed by scRNA-seq and CellChat analyses (human DFU clinical specimens). They also regulate vascular growth by releasing proteases (e.g., MMPs) and cytokines such as TNF-α, VEGF, and TGF-β, which stimulate fibroblast proliferation and ECM production (59). Specific macrophage subsets, such as Lyve1^+^ perivascular macrophages, represent pro-resolving-like cells that maintain tissue integrity, whereas MHCII^hi^ macrophages function as classical pro-inflammatory subsets (human DFU clinical specimens). Lyve1^+^MHCII^lo^ macrophages further regulate vascular homeostasis through activation of the GAS6–AXL signaling pathway (60), potentially influencing angiogenic responses in DFUs (50) (human DFU clinical specimens).

Metabolic dysregulation in diabetes not only alters cytokine production in macrophages but also impairs phagocytosis and wound healing functions (STZ and db/db diabetic mice). Macrophage efferocytosis of apoptotic fibroblasts and vascular cells is essential for resolving inflammation and maintaining homeostasis during early wound repair (47, 48) (human DFU clinical specimens). The transition from pro-inflammatory to pro-resolving macrophages marks the shift from the inflammatory to the proliferative phase of healing. Pro-resolving macrophages facilitate fibroblast proliferation, migration, and differentiation into myofibroblasts, thereby accelerating wound contraction and ECM deposition. Therapeutic interventions can modulate this polarization: the AMPK activator metformin promotes pro-resolving polarization and improves repair (51), while Loureirin B (LB) enhances fibroblast ECM synthesis through TGF-β/Smad signaling (49) (db/db mice). Additionally, the ECM protein asporin suppresses TLR2/4-induced NF-κB activation and cytokine release, mitigating macrophage-driven inflammation in diabetic mouse models. Conversely, the combined effects of hyperglycemia and hypoxia exacerbate macrophage dysfunction through TLR4 signaling, further impairing DFU healing (56) (combined validation of human clinical samples and STZ mice). Importantly, macrophage depletion delays early wound closure but reduces excessive scar formation in later stages, underscoring their context-dependent roles (61). This bidirectional effect is an important source of heterogeneity among current studies (db/db, STZ mice).

With the aid of scRNA-seq, macrophage heterogeneity and signaling networks in DFU wounds can be analyzed at the cellular-subset level. Several macrophage-specific pathological signaling pathways that are difficult to identify using conventional sequencing have been characterized. In DFU lesions, abnormal complement pathway activation may drive macrophages toward a pro-inflammatory phenotype, inhibit pro-resolving polarization, and maintain chronic wound inflammation (62) (human DFU clinical specimens). High expression of the SPP1-mediated inflammatory matrix-interaction pathway may inhibit angiogenesis and ECM remodeling through abnormal intercellular signaling, thereby delaying repair (62) (human DFU clinical specimens). Monocyte subsets can activate a CCL2–CCR2 inflammatory positive-feedback loop, continuously recruit pro-inflammatory immune cells, and engage downstream NLRP3 and S100A8/A9 signaling, thereby amplifying local inflammatory injury. Excessive activation of the cPLA2/COX-2/PGE2 inflammatory lipid pathway in macrophages can further stabilize the pro-inflammatory phenotype through NF-κB signaling and disrupt the normal resolution of inflammation (57) (db/db mice). scRNA-seq has also detected abnormal osteoclast-like macrophage differentiation in diabetic foot ulcer wounds. This subset may activate bone-resorption-related signals and interfere with soft tissue repair, representing a pathological mechanism that conventional sequencing cannot readily capture (61). In addition, disruption of macrophage ribosomal-protein pathways, *CDK2AP2*-mediated cell-cycle arrest, and activation of senescence pathways may cause macrophage functional aging, reduced pathogen-clearance capacity, and sustained inflammatory-factor release, ultimately contributing to a persistent, hard-to-heal wound microenvironment (63) (diabetic mouse wound models).

Overall, macrophages represent a core target for regulating DFU inflammation and tissue repair. Single-cell sequencing provides a powerful approach for analyzing macrophage heterogeneity and intercellular signaling networks. Future large-sample, multi-center, longitudinal clinical studies and animal models that more closely resemble clinical DFU are needed to validate the clinical relevance of these pathways and provide high-quality evidence for macrophage-targeted immunotherapy and vascular regeneration therapy in DFU.

#### 3.1.2 T cells

In addition to macrophages, T lymphocytes also play an important role. T cells are central mediators of immune responses and play multifaceted roles in both immune regulation and tissue repair during wound healing. In DFU, damage to T cell function can cause delayed repair and exacerbate disease severity (human DFU clinical specimens, db/db mice). scRNA-seq enables unbiased identification of T cell subsets, revealing their heterogeneity and functional diversity in DFUs. For example, Vu et al. demonstrated a marked increase in naïve T cells and CD8^+^ T cells in DFU, further resolving 10 naïve T-cell and 7 CD8^+^ T-cell subclusters, underscoring their heterogeneity and therapeutic potential (64) (human DFU clinical specimens).

Functional analyses show that hyperglycemia induces aberrant activation and impaired function of T cells, resulting in dysregulated inflammation and delayed healing (65). Cheng et al. reported significant upregulation of *IL-17* signaling genes in CD8^+^ T cells in DFU, suggesting an enhanced innate barrier function against pathogens (human DFU clinical specimens). Conversely, reduced expression of regulatory genes such as *TSC22D3* and *PIK3IP1* drives excessive CD8^+^ T-cell activation, exacerbating local inflammation (66). An increased proportion of GZMA^+^ CD8^+^ T cells may further contribute to chronic inflammation through the release of granzyme A and IFN-γ (67) (human DFU clinical specimens).

Tregs normally suppress inflammation through IL-10 and TGF-β secretion, inhibition of Th1/Th17 activation, and suppression of pro-inflammatory macrophages (56) (human normal skin wound control specimens). However, scRNA-seq combined with immune infiltration analysis revealed that Tregs are significantly reduced in DFU compared with normal wounds, impairing their anti-inflammatory capacity and promoting Th1/Th17-driven inflammation (56) (human DFU clinical specimens). Similarly, natural killer T cells are enriched in DFUs, where they contribute to inflammation by secreting IFN-γ and IL-4. Tan et al. and Theocharidis et al. reported that DFU samples show high expression of NKT-associated genes, including *NKG7*, *GNLY*, *CCL5*, and *KLRD1*, reinforcing the pathogenic cycle of infection, inflammation, and impaired repair (56, 68) (human DFU clinical specimens). γδ T cells are also increased in diabetic foot ulcer wounds, as shown by Nichols and Pham, and their cytokine release contributes to synergistic inflammatory responses in hyperglycemic environments (50) (aged db/db diabetic mice).

Beyond classical subsets, novel T cell populations have been identified. Naïve T_RGCC cells, enriched in DFUs, regulate MAPK and TCR signaling pathways, highlighting their role in antigen presentation and inflammatory cascades (64) (human DFU clinical specimens). Cytotoxic CTL_TYROBP_CL4 cells, specifically enriched in DFUs, activate NOD-like receptor and TCR signaling pathways, modulating transcription factors such as ZNF597 and RARA, and potentially driving tissue damage.

T cells also influence angiogenesis in DFUs. γδ T cells promote macrophage polarization from pro-inflammatory to pro-resolving via IL-17 secretion, with pro-resolving macrophages subsequently releasing VEGF to stimulate angiogenesis (69) (db/db mice). However, other studies show suppressed Th1/Th17 differentiation in DFUs, leading to inhibition of *IL-17* signaling and impaired endothelial immune regulation, thereby limiting angiogenesis (human DFU clinical specimens).

Overall, single-cell sequencing has greatly enriched our understanding of T-cell heterogeneity, signaling networks, and immune-cell interactions in DFU. Future studies should include longitudinal, large-sample, multi-center clinical cohorts and optimized animal models to further validate the translational value of T-cell-related targets and provide high-quality evidence for precise T-cell-targeted immunotherapy in DFU.

#### 3.1.3 Other immune cells

Neutrophils are essential immune cells in the early phases of wound healing and play a pivotal role in the pathophysiology of DFUs. Recent single-cell sequencing studies have revealed remarkable heterogeneity among neutrophils in DFU tissues, identifying *Retnlg^+^, Lcn2^+^, Wfdc21^+^, and Mmp8^+^* neutrophils (40). Retnlg^+^ and Lcn2^+^ subsets were significantly enriched during the early stages of ulcer formation but markedly decreased during healing, suggesting their involvement in initiating and maintaining inflammatory responses (61). Altered neutrophil abundance not only disrupts their own function but also affects interactions with macrophages, endothelial cells, and fibroblasts, thereby impairing wound healing. Moreover, single-cell transcription-factor network analyses have highlighted dysregulated expression of regulators such as Lcn2, which contributes to neutrophil dysfunction and further delays tissue repair.

**Figure 4:**
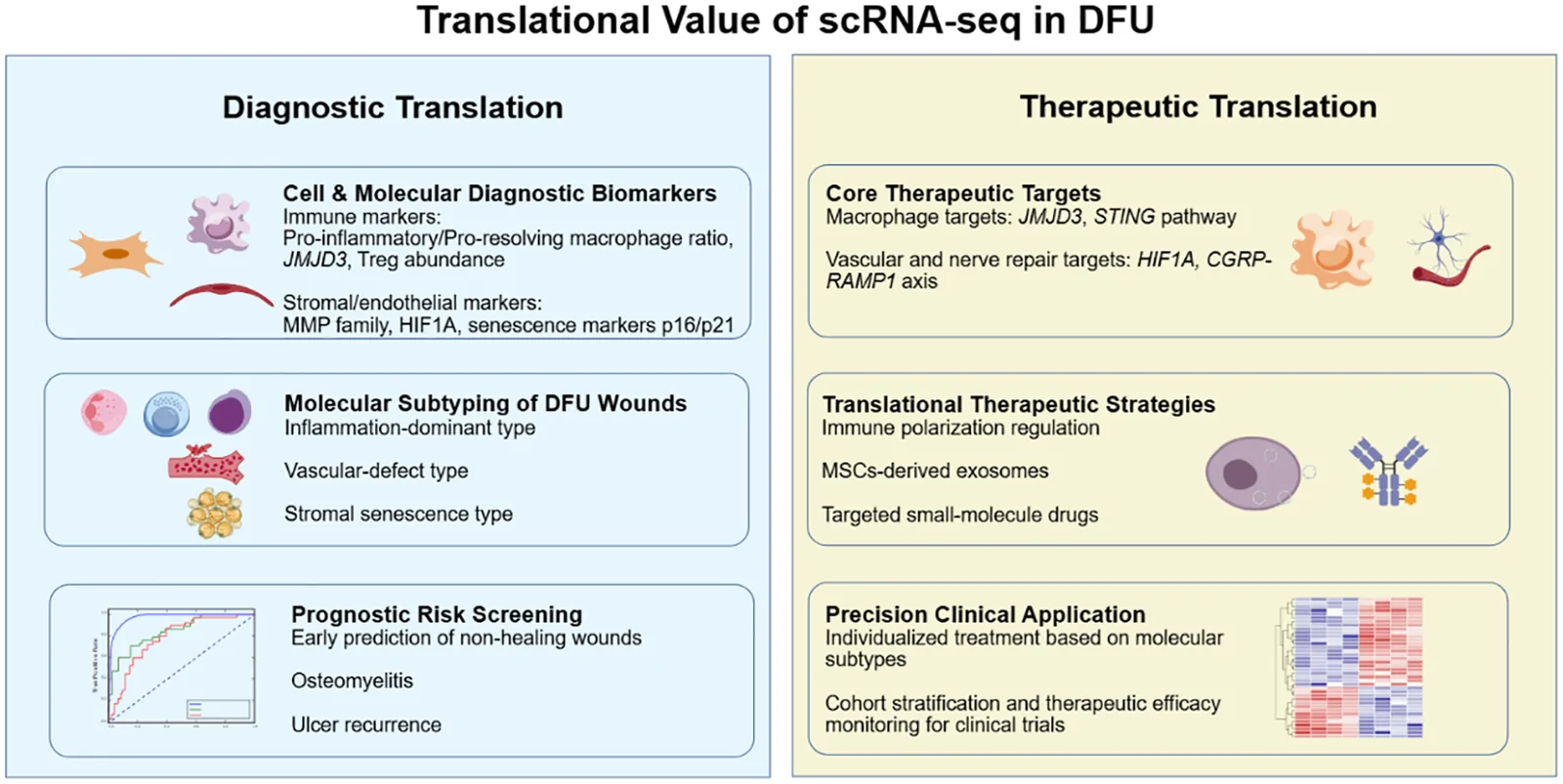
Translational and clinical value of scRNA-seq in dissecting pathogenesis and developing targeted therapy for DFU. This schematic systematically summarizes the translational application framework of single-cell RNA sequencing (scRNA-seq) in diabetic foot ulcer (DFU) research, and is divided into two core modules: diagnostic translation and therapeutic translation. The left panel illustrates multi-layered diagnostic applications supported by scRNA-seq, including cellular and molecular diagnostic biomarkers, molecular subtyping of DFU wounds, and prognostic risk screening for ulcer non-healing, osteomyelitis and recurrence. The right panel elaborates therapeutic translational prospects, covering core therapeutic targets derived from single-cell transcriptome data, multiple translational intervention strategies, and individualized precision clinical treatment based on molecular subtypes. Collectively, this figure comprehensively expounds how scRNA-seq bridges basic wound mechanism research and clinical precision diagnosis and treatment of DFU. Created with BioGDP.com.

Dendritic cells (DCs), as professional antigen-presenting cells, are equally important in orchestrating immune responses and tissue repair in DFUs. scRNA-seq studies have identified increased numbers of classical DC subsets expressing MHC class II molecules (e.g., *H2-Ab1, H2-Aa, H2-Eb1*), as well as migratory DCs characterized by CCR7, FSCN1, CCL22, and TBC1D4 (70). These subsets are implicated in recruiting immune cells and coordinating local responses. Importantly, DCs rely on communication with macrophages, fibroblasts, and endothelial cells to modulate wound healing. Mechanistically, loss of the transcription factor FOXM1 in DFU-associated DCs was linked to reduced neutrophil recruitment, while activation of TREM1 enhanced FOXM1-mediated pathways and promoted wound repair (70) (**Table 1**) (**Figure 4**).

**Table 1:** Characteristics of major immune cell subpopulations and dysregulated signaling molecules in the DFU wound microenvironment.

Cell type	Core subpopulations	Key characteristics	Regulatory pathways/molecules with expression trend	Sample source	Replication & data origin description
Macrophages	pro-inflammatory	Pro-inflammatory, secrete TNF-α/IL-1β, high expression of *IL1B/S100A8/S100A9*	TNF-α ↑, IL-1β ↑ (51), NF-κB signaling ↑ (53–55)(hyperactivated)	Human DFU clinical specimens, STZ-induced type 1 diabetic mice, db/db type 2 diabetic mice	Multi-human cohort validation, supplemented with independent mouse model supporting data
	pro-resolving	Anti-inflammatory, promote tissue remodeling, high expression of *C1QA/B/C/CD163*	*VEGFA* ↓, TGF-β ↓, AMPK signaling ↓ (59) (inhibited)	Human DFU clinical specimens, db/db mice	Multi-human cohort validation, verified in db/db diabetic mice
	Special Subpopulations	Lyve1^+^MHCII^lo^ (maintain vascular homeostasis), MHCII^hi^ (pro-inflammatory)	GAS6-AXL signaling activated↑ (60)	Human DFU clinical specimens	Single human dataset only, no external patient cohort replication
T Cells	Naive T Cells	10 subpopulations, significantly increased in number	Not reported	Human DFU clinical specimens	Single human dataset only
	CD8^+^T Cells	7 subpopulations, excessive activation, secretion of GZMA/IFN-γ, and high expression of IL-17 signaling genes	IL-17 ↑ (67)	Human DFU clinical specimens	Multi-human cohort validation, consistent results across two independent patient groups
	Other Subpopulations	Tregs (decreased in number), NKT (enriched, secrete IFN-γ/IL-4), γδ T (enriched, secrete IL-17), T_RGCC (enriched), CTL_TYROBP_CL4 (specifically enriched)	MAPK ↑, TCR ↑, NOD-like receptors activated ↑ (64)	Human DFU clinical specimens	Single human dataset; independent validation not reported
Other Immune Cells	Neutrophils	*Retnlg^+^/Lcn2^+^/Wfdc21^+^/Mmp8^+^* subpopulations, enriched in early stage	*Lcn2* ↑ (61)	Human DFU clinical specimens	Single human dataset only
	Dendritic Cells (DCs)	Classical DCs (high expression of MHCII), Migratory DCs (high expression of CCR7/FSCN1)	*FOXM1* ↓, *TREM1* ↑ (70)	Human DFU clinical specimens	Single human dataset only, supported by *in vitro* dendritic cell functional tests

↑ represents significantly upregulated expression or pathway activation; ↓ represents significantly downregulated expression or pathway inhibition.

DFU, diabetic foot ulcer; TNF-α, tumor necrosis factor-α; IL-1β, interleukin-1β; NF-κB, nuclear factor kappa-B; VEGFA, vascular endothelial growth factor A; TGF-β, transforming growth factor-β; AMPK, adenosine 5’-monophosphate-activated protein kinase; Lyve1, lymphatic vessel endothelial hyaluronan receptor 1; MHCII, major histocompatibility complex class II; GAS6, growth arrest-specific gene 6; AXL, AXL receptor tyrosine kinase; GZMA, granzyme A; IFN-γ, interferon-γ; IL-17, interleukin-17; Tregs, regulatory T cells; NKT, natural killer T cells; MAPK, mitogen-activated protein kinase; TCR, T cell receptor; NOD, nucleotide-binding oligomerization domain; Lcn2, lipocalin 2; DCs, dendritic cells; CCR7, C-C chemokine receptor type 7; FSCN1, fascin actin-bundling protein 1; FOXM1, forkhead box M1; TREM1, triggering receptor expressed on myeloid cells 1.

#### 3.1.4 Current limitations of scRNA-seq studies of DFUs

The key pathological basis for the persistence of diabetic foot ulcers is the imbalance of macrophage polarization, the defect in the repair function of pro-resolving macrophages, and the abnormal activation of inflammatory pathways (71). However, existing research conclusions still exhibit heterogeneity and academic disputes. Firstly, there is no unified theory regarding the correlation between the total number of macrophages infiltrating wounds and healing prognosis. The fundamental reason lies in the fact that most studies have not strictly divided the stages of ulcer disease, nor have they distinguished between different functional subpopulations of macrophages. Secondly, macrophage knockout interventions have yielded bidirectional research results, primarily due to different intervention time points. Additionally, there are significant differences in the pathogenic value of osteoclast-like macrophages between simple skin ulcers and wound samples accompanied by osteomyelitis (56). Simultaneously, the traditional pro-inflammatory/pro-resolving binary polarization classification model is overly simplified and does not match the heterogeneity of macrophage lineages revealed by single-cell sequencing, leading to discrepancies between older and newer findings (61).

T-cell immune dysregulation is the basic consensus on the pathogenesis of DFU. Hyperglycemia-mediated abnormal activation of T cells, excessive inflammatory infiltration of CD8^+^ T cells, natural killer T cells, and γδ T cells, as well as functional defects of CD8^+^ T cells, are important immune mechanisms underlying chronic inflammation in DFU. However, there remains significant heterogeneity and controversy. These mainly involve bidirectional upregulation or downregulation of IL-17 signaling, dual pro-inflammatory and repair functions of the same T cell subsets, and insufficient reproducibility of novel rare T cell subsets. These controversies may be caused by inconsistent wound staging, infection status, glycemic control levels, and analysis criteria. In addition, pathways involving novel T cell subsets are mostly based on bioinformatics predictions, and the causal mechanisms are unclear.

From the perspectives of methodology and evidence quality, existing basic research exhibits several limitations: Firstly, DFU experiments typically utilize db/db mice and STZ-induced diabetes mouse models. However, these studies are often single-center and based on small sample sizes. These models cannot spontaneously develop chronic foot ulcers that fully reproduce human diseases, nor can they fully simulate complex clinical conditions such as peripheral neuropathy, peripheral arterial disease, local hypoxia, and mixed infections. This makes it difficult to directly extrapolate the treatment effects from animal experiments to clinical populations (72). Additionally, human DFU is characterized by persistent chronic inflammation, aging macrophage function, impaired polarization conversion, high amputation risk, and high mortality, while diabetic mice mainly exhibit delayed wound inflammation without comparable long-term adverse prognosis phenotypes (73). Clinical tissue sampling and single-cell sequencing studies also face design limitations, including limited case numbers, inadequate ulcer stratification, and non-standardized control settings, which restrict the generalizability of research results (61). Existing research is mostly cross-sectional and static observation, lacking dynamic temporal verification. It focuses on gene and protein expression changes, but lacks *in vitro* functional experiments and clinical prognostic correlation analysis. Epigenetics and inflammation-lipid regulation pathways are still in the basic mechanistic stage, requiring extensive verification before achieving clinically targeted immunotherapy (71).

### 3.2 Extracellular matrix remodeling and tissue repair mechanisms

#### 3.2.1 Fibroblast heterogeneity and tissue remodeling

Fibroblasts are central players in wound repair and host defense during DFUs, and their functional integrity is essential for both infection control and tissue regeneration (**Figure 3**). Characterizing fibroblast heterogeneity, delineating subpopulations and lineages, and understanding their interactions with immune cells are critical for developing strategies to accelerate wound healing and reduce the clinical burden of chronic ulcers (74) (**Figure 4**).

scRNA-seq has been instrumental in defining fibroblast heterogeneity in human skin. Buechler et al. generated a single-cell fibroblast atlas, classifying lineages into general steady-state cells (DPT^+^), specialized steady-state cells, and disturbance-specific activated cells. This revealed the remarkable diversity and plasticity of fibroblasts, offering new therapeutic perspectives for DFU management (74). Similarly, Januszyk et al. compared fibroblasts from diabetic and non-diabetic ulcers and across different ethnic groups, uncovering distinct subpopulations with varied fibrotic potentials and transcriptional programs in human DFU clinical samples (75). These subtypes expressed genes linked to immune modulation and ECM remodeling, suggesting that pro-healing fibroblast subsets may serve as key therapeutic targets (56, 58).

Mechanistic insights have also emerged. Ku et al. showed that dysregulation of the *IGF-1–SP1–CD248* pathway impairs fibroblast migration and delays healing in diabetic foot ulcer wounds. Crosstalk between fibroblasts, endothelial cells, T cells, and tissue stem cells is mediated through CCL signaling, which is elevated in healing compared with non-healing human DFU tissue (76). Healing-associated fibroblasts display enhanced ECM remodeling and regenerative capacity, whereas non-healing fibroblasts exhibit features of cellular senescence and complement activation in human DFU lesions, underscoring the importance of fibroblast–immune cell interactions in wound outcomes (75).

Although scRNA-seq has greatly advanced our understanding of fibroblast transcriptional heterogeneity in DFUs, it cannot capture the native spatial distribution and regional functional differences of fibroblast subsets in intact ulcer tissues. Recent spatial transcriptomics studies have further complemented and deepened this perspective, revealing spatially programmed fibroblast zonation that fundamentally determines wound repair outcomes. In human DFU tissues, pro-healing fibroblast subsets characterized by high expression of *MMP1*, *MMP3*, and *TNFAIP6* are predominantly enriched in the central granulation bed, where they drive ECM remodeling and angiogenesis to facilitate tissue regeneration. In contrast, senescent and pro-inflammatory fibroblast populations mainly accumulate at the wound margin and avascular ulcer base, continuously sustaining chronic inflammatory microenvironments and blocking wound closure (56).

Spatial cell communication analysis further demonstrates that spatially segregated fibroblast subsets form distinct immune crosstalk patterns in different ulcer regions. Regenerative fibroblasts in granulation tissue interact with pro-resolving macrophages to promote inflammation resolution and tissue reconstruction, while senescent fibroblasts at the wound edge induce persistent pro-inflammatory macrophage activation, thereby maintaining a non-healing state (77). Collectively, emerging spatial transcriptomics evidence indicates that the dysfunctional spatial distribution of fibroblast subpopulations, rather than simple transcriptional alterations, is a core determinant of refractory diabetic foot ulcer progression.

#### 3.2.2 Vascular endothelial dysfunction inhibits wound angiogenesis

Single-cell transcriptomic data consistently demonstrate remarkable subpopulation diversification and functional dysregulation of endothelial cells in diabetic foot ulcer wounds. In healthy skin, endothelial cells steadily secrete pro-angiogenic factors such as VEGF, FGF1 and FGF2, which coordinately regulate endothelial proliferation and tube formation to enable rapid reconstruction of the wound microcirculation. By contrast, endothelial cells within DFU lesions globally exhibit senescent and hypoproliferative phenotypes, accompanied by widespread suppression of pro-angiogenic signaling pathways. Integrated analysis of the public dataset GSE165816 reveals that core genes including *CCND1, ENO1, HIF1A* and *SERPINE1* are significantly downregulated in endothelial cells of DFU wounds. These genes are involved in cell cycle regulation, hypoxic response, vascular remodeling and microcirculatory homeostasis maintenance, respectively. Their dysregulated expression directly causes defective microvascular neogenesis in the lower extremities and insufficient blood and oxygen supply to local tissues, giving rise to a persistent ischemic and hypoxic microenvironment that further amplifies the blockade of wound repair (78).

Single-cell atlases have further identified two characteristic endothelial cell subsets: functionally reparative endothelial cells with high expression of *SLCO2A1* and *CYP1A1*, and senescent dysfunctional endothelial cells. In the granulation tissue of wounds with favorable healing potential, reparative endothelial cells are highly enriched and form a positive reparative circuit with pro-resolving macrophages and regenerative fibroblasts via ligand-receptor interactions. In refractory ulcer wounds, by contrast, senescent endothelial cells predominate, with markedly impaired migratory and proliferative capacities; meanwhile, they secrete abundant anti-angiogenic factors such as TSP1 and PEDF, which persistently counteract the angiogenic process (56).

At the pathway regulation level, multiple key signaling pathways in endothelial cells, including PI3K/Akt, MAPK, Notch, Wnt/β-catenin and Nrf2, undergo aberrant suppression. The dual stimulation of hyperglycemia and hypoxia blocks the Nrf2 antioxidant pathway, exacerbating oxidative stress damage in endothelial cells. Persistent activation of the NF-κB pathway, in turn, induces endothelial cells to secrete large quantities of pro-inflammatory mediators and amplifies local inflammatory responses, forming a vicious cycle of endothelial injury, microcirculatory ischemia and chronic inflammation-driven endothelial senescence (79).

In addition, dysregulated intercellular communication between endothelial cells, macrophages and fibroblasts further aggravates angiogenic impairment. Senescent endothelial cells fail to effectively release VEGFA to engage VEGFR1 on the macrophage surface, which impedes macrophage polarization toward the pro-resolving phenotype and indirectly inhibits extracellular matrix remodeling and granulation tissue growth (77).

#### 3.2.3 Functional characteristics of epidermal keratinocytes in re-epithelialization

Beyond fibroblasts, epidermal cells play an indispensable role in reepithelialization. Liu et al. identified diabetes-associated keratinocyte subsets, including BC-2 cells, which remain inactive in human DFU tissues and impair epidermal differentiation (67). In contrast, keratinocyte subsets enriched in well-healed wounds express VEGF and KGF, promoting angiogenesis, fibroblast proliferation, and collagen synthesis through paracrine mechanisms in human and diabetic mouse models. Conversely, poorly healing wounds are enriched with epidermal cells expressing senescence-related markers such as p16INK4a and p21Cip1 in both clinical samples and animal models, resulting in loss of proliferative and migratory capacity (47) (**Figure 4**).

Integrated single-cell multi-omics analyses further delineate the heterogeneous regulatory network of keratinocytes: during normal wound repair, keratinocytes activate pro-reparative pathways including TGF-β/Smad and MAPK, whereas in the hyperglycemic microenvironment, persistent overactivation of the TLR4/NF-κB pathway suppresses keratinocyte differentiation and induces cellular senescence (67).

Meanwhile, keratinocytes engage in extensive crosstalk with macrophages and endothelial cells. Cytokines secreted by reparative keratinocytes promote macrophage polarization toward the anti-inflammatory pro-resolving phenotype, while senescent keratinocytes sustain the release of TNF-α and IL-1β from macrophages, exacerbating inflammatory damage in the wound bed (54).

Targeted intervention strategies against keratinocyte senescence and differentiation defects, such as activating the Nrf2 antioxidant pathway or inhibiting senescence-associated signaling, can effectively restore the reparative function of keratinocytes and provide novel therapeutic insights for promoting wound re-epithelialization (80).

#### 3.2.4 Sensory neurons mediate neuro-immune coordinated wound repair

Neurons also contribute to DFU tissue repair. Lu et al. demonstrated that nociceptive neurons, particularly those expressing calcitonin gene-related peptide (CGRP), coordinate immune responses during wound healing. Loss of NaV1.8^+^ sensory neurons impairs skin closure and muscle regeneration. CGRP released from neuronal terminals engages *RAMP1* on neutrophils, monocytes, and macrophages in mouse *in vivo* and *in vitro* cellular experiments, reducing recruitment, accelerating apoptosis, enhancing phagocytosis, and polarizing macrophages toward pro-resolving phenotypes, thereby promoting healing (81) (**Table 2**).

**Table 2:** Biological features and dysregulated regulatory molecules of fibroblasts, epidermal cells and neurons in DFU wound microenvironment.

Cell type	Core subpopulations/phenotypes	Key characteristics	Regulatory pathways/molecules with expression trend	Sample source	Replication & data origin description
Fibroblasts	Healing-related Type	High expression of *MMP1/3/11/HIF1A*, enhanced ECM remodeling	CCL signaling ↑ (76)	Human DFU clinical samples	Multi-human cohort validation across multiple independent patient groups
	Non-healing-related Type	Cellular senescence, complement activation	*IGF-1–SP1–CD248* axis dysregulated ↓ (76)	Human + Mouse	Multi-human cohort validation, functional verification in db/db mice
Vascular Endothelial Cells	Senescent hypoproliferative phenotype; impaired angiogenesis; pro-inflammatory activation; two subsets: reparative (SLCO2A1+/CYP1A1+) and senescent dysfunctional endothelial cells	Downregulated: *CCND1, ENO1, HIF1A, SERPINE1*; Reparative markers: *SLCO2A1, CYP1A1*; Anti-angiogenic factors: TSP1, PEDF	PI3K/Akt, MAPK, Notch, Wnt/β-catenin, Nrf2 ↓ NF-κB ↑ (79)	Human DFU specimens, db/db mice, STZ-induced diabetic mice	Verified in human cohorts and multiple animal models
Epidermal Cells	BC-2 Subtype	Quiescent state, impaired differentiation	Not reported	Human DFU clinical specimens	Single human scRNA-seq dataset; independent validation not reported
	Healing-related Type	Secrete VEGF/KGF, promote angiogenesis/collagen synthesis	VEGF ↑, KGF ↑ (47)	Human + Mouse	Multi-human cohort validation, confirmed via *in vivo* mouse rescue experiments
	Senescent Type	High expression of p16INK4a/p21Cip1, loss of proliferation/migration capacity		Human + Mouse	Multi-human cohort validation of cellular senescence molecular signatures
Neurons	Nociceptive Neurons	Express *CGRP/NaV1.8^+^*, regulate immune cell function	CGRP–RAMP1 signaling ↑ (81)	Human + Mouse	Single human single-cell nerve dataset, validated by mouse neural immune functional assays

↑ represents significantly upregulated expression or pathway activation; ↓ represents significantly downregulated expression or pathway inhibition.

DFU, diabetic foot ulcer; ECM, extracellular matrix; CGRP, calcitonin gene-related peptide; RAMP1, receptor activity-modifying protein 1; IGF-1, insulin-like growth factor 1; SP1, specificity protein 1; VEGF, vascular endothelial growth factor; KGF, keratinocyte growth factor; Nrf2, nuclear factor erythroid 2-related factor 2; TSP1, thrombospondin 1; PEDF, pigment epithelium-derived factor.

**Figure 5:**
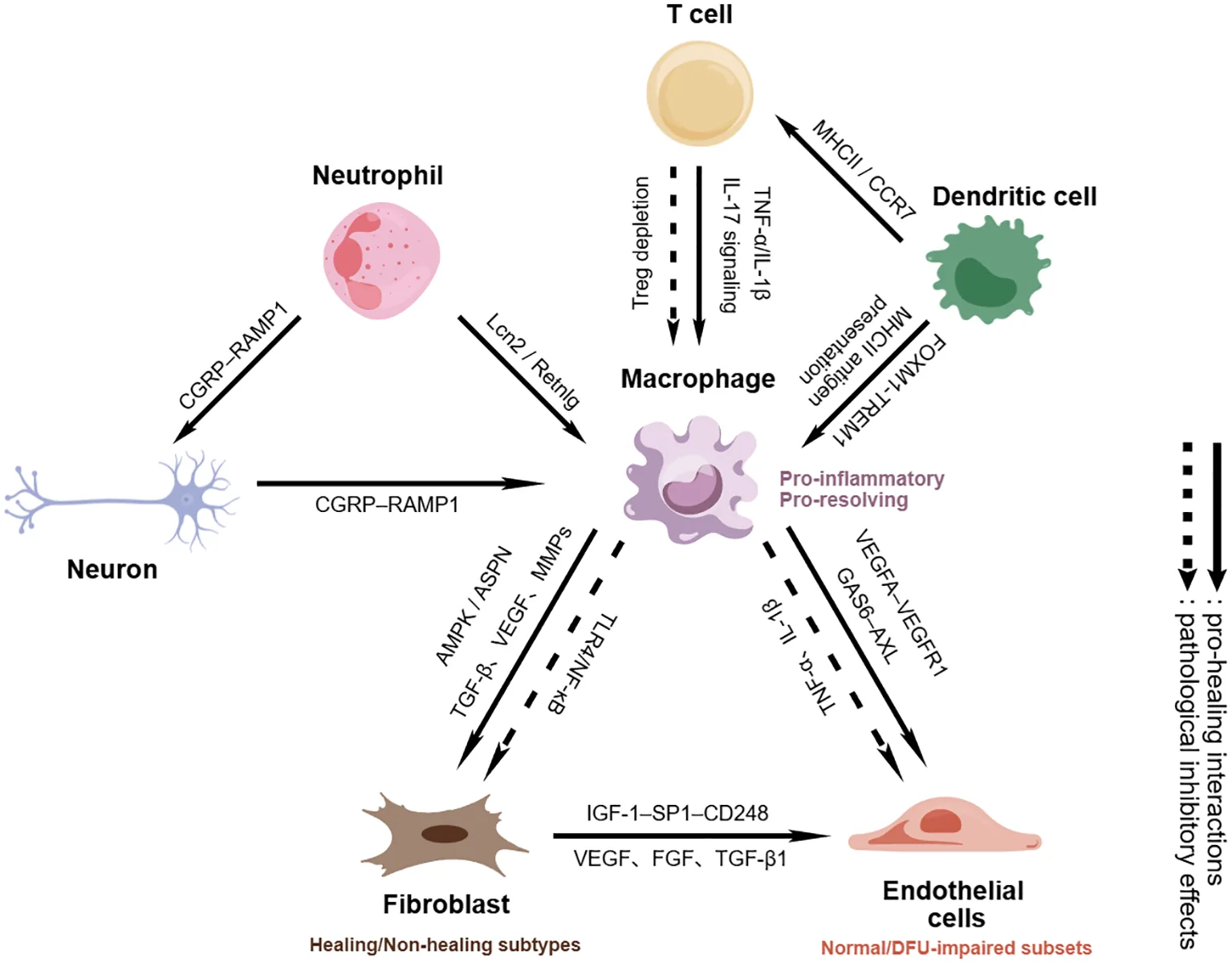
Multi-cell crosstalk network in DFU wound microenvironment uncovered by scRNA-seq. Macrophages act as the central hub of wound immunity. Solid black lines indicate pro-healing ligand-receptor interactions between neurons, T cells, dendritic cells, neutrophils, fibroblasts and endothelial cells; dashed lines mark pathological signal transmission that aggravates inflammation and blocks tissue repair. Created with BioGDP.com.

Collectively, scRNA-seq has revealed the heterogeneity and specialized roles of fibroblasts, epidermal cells, and neurons in DFUs. By uncovering cellular communication patterns and molecular networks among these populations and their interactions with immune cells, single-cell approaches provide a comprehensive framework for understanding tissue repair mechanisms and offer critical insights for developing targeted therapies to enhance wound healing (75) (**Figures 4**, **5**).

### 3.3 Exploration of therapeutic targets and biomarkers

DFU is one of the most serious complications of diabetes and is characterized by complex non-healing mechanisms, high rates of disability, amputation, and mortality, and limited efficacy of conventional treatments. Therefore, identifying new biomarkers and developing targeted therapeutic strategies are urgent clinical priorities (82). scRNA-seq technology overcomes the averaging limitation of conventional bulk sequencing and enables high-resolution analysis of the DFU wound microenvironment at single-cell resolution. By deciphering cellular heterogeneity, abnormal signaling pathways, and intercellular communication networks, scRNA-seq substantially accelerates the screening of prognostic biomarkers, potential therapeutic targets, and candidate drugs for translational DFU research (61) (**Figure 4**).

Multiple single-cell and multi-omics studies have shown that fibroblasts and macrophages are two key stromal and immune effector-cell populations regulating DFU healing (66). In DFU wounds with favorable healing tendencies, pro-resolving macrophage subsets are enriched, alongside fibroblast subsets highly expressing *MMP1*, *MMP3*, *MMP11*, *HIF1A, CHI3L1*, and *TNFAIP6*. These fibroblast subsets sequentially mediate ECM remodeling, stress tolerance, inflammatory regulation, and tissue repair and are considered key cellular components driving normal healing in diabetic foot ulcer wounds (56). Beyond these repair-related gene signatures, DFU-derived fibroblasts exhibit impaired migration and proliferation together with increased cellular senescence, providing a cellular basis for defective wound repair (83).

Spatial transcriptomics and scRNA-seq validate and complement each other, further confirming the decisive role of immune-cell distribution and functional abnormalities in DFU. Spatial mapping further indicated that wounds with favorable healing were characterized by resolution of the inflammatory phase and a shift toward pro-resolving macrophage states. In contrast, refractory non-healing ulcers showed persistent accumulation of pro-inflammatory macrophages and impaired transition toward pro-resolving states, resulting in sustained inflammation, defective granulation tissue formation, and delayed wound closure. These findings highlight the importance of macrophage-state transitions and local immune homeostasis in determining DFU healing outcomes (56).

In recent years, scRNA-seq has continuously revealed molecular mechanisms underlying macrophage dysfunction, providing a theoretical basis for target development. Single-cell transcriptomic analysis of the JMJD3 regulatory network showed that the epigenetic regulator JMJD3 can activate the JAK1/3–STAT3 signaling axis and amplify NF-κB-mediated release of inflammatory cytokines, including tumor necrosis factor (TNF) and interleukins, thereby trapping macrophages in a pro-inflammatory state and delaying inflammation resolution (57). Therefore, JMJD3 may serve as a potential therapeutic target for reprogramming macrophage phenotypes and suppressing chronic inflammation. Excessive activation of the stimulator of interferon genes (STING) pathway is also closely associated with persistent inflammation in late-stage DFU. Abnormal activation of this pathway can amplify innate immune inflammation and disrupt the temporal sequence of wound healing, making it a potentially valuable candidate target for DFU intervention. In addition to immune-cell abnormalities, endothelial-cell dysfunction is another core factor contributing to vascular defects, inadequate local blood supply, and impaired healing in DFU (61). Integrated analysis of the GSE165816 public dataset and diabetic-wound transcriptomic data revealed that *CCND1, ENO1, HIF1A, SERPINE1*, and other genes involved in cell-cycle metabolism, hypoxia response, angiogenesis, and immune regulation were significantly downregulated. Imbalanced expression of these core genes may directly cause lower-limb microvascular neogenesis defects, insufficient microcirculatory perfusion, and tissue hypoxia/ischemia, thereby establishing a vascular pathological basis for DFU (78) (**Figure 4**).

Building an integrated drug-target regulatory network based on scRNA-seq transcriptome data can efficiently screen disease core molecular nodes and potential therapeutic drugs. Through this analysis strategy, existing research has successfully identified seven potential core therapeutic targets for DFU and 20 candidate small-molecule drugs with clinical translational application potential, providing data support and theoretical reference for subsequent basic experimental verification and clinical targeted drug development (84). On the whole, single-cell and spatial multi-omics technology can accurately depict cellular heterogeneity and molecular pathological network of DFU microenvironment, which can both explain the mechanisms of disease onset and progression and screen reliable diagnostic biomarkers and intervention targets, opening up a new research direction for precise targeted treatment of diabetic foot ulcers (65) (**Figures 3**, **4**) (**Table 3**).

**Table 3:** Candidate cellular biomarkers and their biological characteristics in the DFU wound microenvironment.

Biomarker	Cell source	Expression trend (DFU vs. normal)	Core significance	Functional validation & translational potential	Replication & data origin description
*MMP1*	Healing-associated fibroblasts	↑ (56)	Indicator of ECM remodeling and wound repair	Preclinical evidence; candidate biomarker for evaluating fibroblast reparative activity in DFU	Multi-human cohort validation across ≥3 independent DFU patient groups
*MMP3*	Healing-associated fibroblasts	↑ (56)	Indicator of extracellular matrix turnover	Correlated with inflammatory severity; potential marker for inflammatory stratification	Multi-human cohort validation
*MMP11*	Healing-associated fibroblasts	↑ (56)	Signature of pro-resolving fibroblast subsets	Closely related to stromal remodeling; available for assessing wound regenerative capacity	Multi-human cohort validation + *in vitro* fibroblast functional verification
*HIF1A*	Healing-associated fibroblasts, Endothelial cells	↓ (78)	Indicator of hypoxia response and tissue repair regulation	Downregulation aggravates ischemic-hypoxic injury; serves as a prognostic biomarker and therapeutic target	Multi-human cohort validation + gene knockout mouse functional proof
*CHI3L1*	Healing-associated fibroblasts	↑ (56)	Indicator of wound inflammatory balance and reparative microenvironment	Reflects fibroblast-mediated repair status; candidate marker for distinguishing healing and non-healing DFU	Multi-human cohort validation with consistent differential expression
*TNFAIP6*	Healing-associated fibroblasts	↑ (56)	Indicator of immune homeostasis and anti-inflammatory repair	Modulates stromal immune crosstalk; predictive biomarker for wound recovery prognosis	Multi-human cohort validation
*CCND1*	Endothelial Cells	↓ (78)	Indicator of angiogenic capacity	Downregulation leads to angiogenesis deficiency; promising therapeutic and predictive biomarker	Single human endothelial single-cell dataset, supplemented with mouse tube formation functional test
*ENO1*	Endothelial Cells	↓ (78)	Indicator of endothelial function status	Endothelial dysfunction related; can serve as an indicator of vascular repair failure	Single human dataset only
*SERPINE1*	Endothelial Cells	↓ (78)	Indicator of vascular remodeling and microcirculation homeostasis	Dysregulation induces vascular homeostasis disorder; prognostic marker for refractory DFU	Human diabetic-wound scRNA-seq with mechanistic validation in diabetic mouse models
*JMJD3*	Macrophages	↑ (52)	Indicator of epigenetic inflammatory activation	Drives sustained chronic inflammation; targetable regulator for macrophage immunotherapy	Human diabetic-wound scRNA-seq with mechanistic validation in diabetic mouse models

↑ represents significantly upregulated expression or pathway activation; ↓ represents significantly downregulated expression or pathway inhibition.

DFU, diabetic foot ulcer; MMP, matrix metalloproteinase; ECM, extracellular matrix; HIF1A, hypoxia inducible factor 1 subunit alpha; CHI3L1, chitinase 3 like 1; TNFAIP6, tumor necrosis factor alpha induced protein 6; CCND1, cyclin D1; ENO1, enolase 1; SERPINE1, serpin family E member 1; JMJD3, jumonji domain containing 3.

## 4 Summary and future outlook

Single-cell transcriptomics advances the exploration of DFU pathogenesis and deepens the understanding of immune disturbance, impaired angiogenesis and disordered tissue remodeling. Combined with computational algorithms, scRNA-seq characterizes functions and intercellular communication of macrophages and T cells, enabling efficient identification of therapeutic targets and biomarkers.

Nevertheless, clinical translation is hindered by technical standardization, multi-omics integration and target validation. Macrophage-enriched JMJD3 drives persistent inflammation and serves as a promising immunomodulatory target. Future work should establish single-cell diagnostic systems, develop markers for microcirculation homeostasis to bridge bench-to-bedside translation, optimize regenerative strategies and expand drug discovery. Single-cell technologies are poised to transform DFU management, facilitate precision medicine and improve patient outcomes (**Figure 4**).

### 4.1 The potential of multi-omics integration in single-cell technology

With rapid technological advances, single-cell research has evolved from focusing on a single transcriptomic layer to embracing multi-omics integration. This includes the combination of spatial transcriptomics, spatial metabolomics, single-cell proteomics (e.g., CyTOF), and single-cell epigenomics. By integrating multimodal data, researchers can construct multidimensional mechanistic maps that span transcriptional regulation, epigenetic control, and cellular communication networks. Such approaches provide deeper systems-level insights into disease biology and enable the identification of critical pathological pathways and therapeutic intervention points.

**Figure 6:**
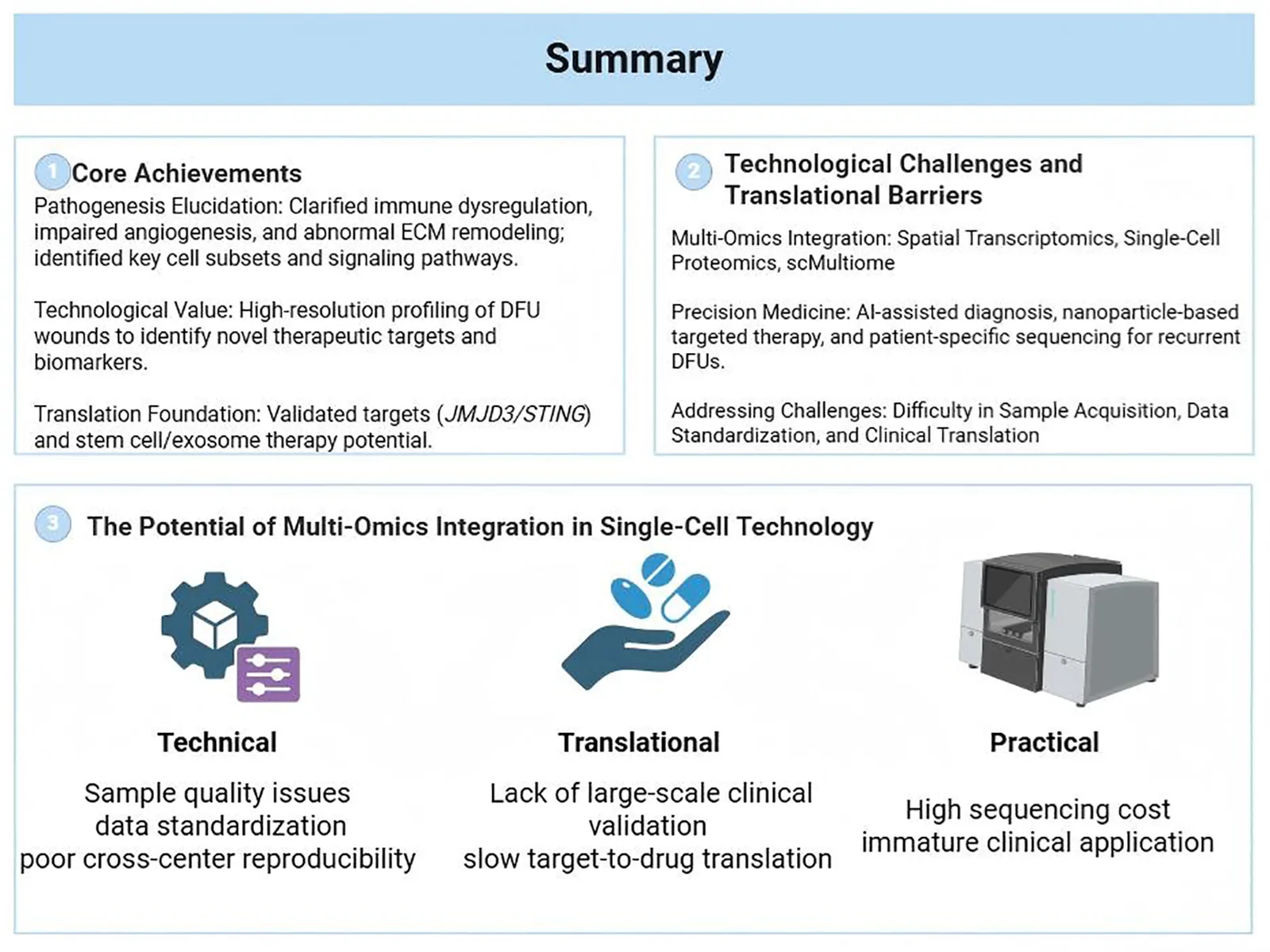
Comprehensive summary of scRNA-seq and multi-omics research on DFU. The graph is divided into three parts: core research achievements including clarified DFU pathological mechanisms and validated therapeutic targets; existing technical and clinical translational barriers; three-layer future prospects of multi-omics integration covering technical, translational and practical clinical limitations. Created with BioRender.com.

In the context of DFUs, preliminary studies have already combined scRNA-seq with spatial localization and immunofluorescence validation, uncovering functional differences between pro-inflammatory/pro-resolving macrophages, endothelial cells, and fibroblast subsets in wound healing (56, 84). Looking ahead, the integration of single-cell multi-omics with joint sequencing technologies promises to deliver a more comprehensive characterization of the DFU microenvironment. These advances will not only enhance mechanistic understanding but also establish the foundation for personalized therapeutic strategies (**Figure 6**).

### 4.2 Technological challenges and translational barriers

This review clarified the imbalance and abnormal signaling of macrophage and T-cell subsets, effectively compensating for the shortcomings of traditional omics research, improving our understanding of the pathological mechanisms of immune dysregulation that impair wound healing in a hyperglycemic environment, and providing a refined theoretical basis for the development of therapeutic targets such as JMJD3 and for molecular subtyping of wounds. It is consistent with previous research on inflammation regulation and vascular repair, and has positive significance for promoting precision diagnosis and treatment of DFU.

However, related research still has several limitations. First, fresh clinical samples are difficult to obtain and sample quality is variable. Moreover, sequencing platforms and bioinformatics pipelines lack unified standards. Therefore, conclusions from small-sample cross-sectional studies are not universally generalizable, and results across studies are difficult to validate. Second, most mechanistic findings remain at the basic-research stage and lack large-sample, multi-center cohort validation and rigorous functional experimental evidence. Differences between human pathology and animal models have also not been fully considered. As a result, translation into clinical diagnosis, treatment, and drug development remains slow. In addition, the high experimental and computational costs of single-cell sequencing further constrain its broad clinical application (38) (**Figure 6**).

Most existing single-cell RNA sequencing studies on diabetic foot ulcers rely on animal models such as STZ-induced type 1 diabetic mice and db/db spontaneous type 2 diabetic mice. However, there are inherent species-specific differences between mice and humans in skin structure, wound microenvironment, immune cell composition, and wound healing kinetics. Mouse skin is thinner with a simpler subcutaneous tissue structure, making it difficult to spontaneously form chronic non-healing ulcers that are highly consistent with clinical manifestations. Additionally, mouse models cannot fully replicate the true pathological features of human DFU, including concurrent peripheral neuropathy, lower extremity arterial occlusion, recurrent mixed infections, and long-term high amputation risk. In these animal models, the polarization patterns of immune subsets such as macrophages and T cells, as well as the kinetics of inflammation resolution, do not completely align with those in human wounds. Differentially expressed genes, signaling pathways, and potential therapeutic targets identified via scRNA-seq in mice are difficult to directly extrapolate to clinical patients. Furthermore, most animal experiments only observe short-term wound healing phenotypes and lack follow-up validation of long-term prognosis, complications, and clinical translation outcomes, leading to a significant evidence gap when translating basic research findings into clinical precision diagnosis and treatment. Future studies should prioritize single-cell sequencing of human DFU clinical wound samples to further narrow the species differences and translational gap between basic research and clinical application.

Overall, the conclusions of this review provide useful references for subsequent in-depth exploration of DFU immune mechanisms, biomarker screening, and clinical trial design. With unified research standards, expanded clinical sample sizes, and reduced technical barriers, these findings may help bridge basic research and clinical practice and support early screening, stratified intervention, and individualized precision treatment of DFUs.

### 4.3 Existing research difficulties

Four major challenges currently limit the application of single-cell sequencing in diabetic foot ulcer research: sample acquisition and standardization, technical limitations, mechanistic uncertainty, and clinical translation. Firstly, at the sample level, the quality control of fresh tissue sampling at DFU is difficult, and there is a lack of unified collection and sequencing standards. Existing studies are mostly small sample cross-sectional studies, and db/db and STZ mouse models cannot replicate the complex lesions of human DFU nerves, blood vessels, and mixed infections, making it difficult to clinically extrapolate the targets. At the technical level, most studies rely on single-omics approaches, resulting in limited multidimensional information. In terms of mechanism, the pro-inflammatory/pro-resolving binary model of macrophages cannot match the true cell lineage. There is bidirectional controversy over the function of T cell subsets, and the regulatory network of the interaction between matrix, endothelium, and nerve cells is not fully elucidated. The cascade pathogenic sequence of high glucose, ischemia, infection, and nerve damage is unclear. Finally, at the translational level, targets such as JMJD3 and STING have only been validated in animals, lacking large-scale human trials and standardized prognostic risk models. Targeted drugs also lack clinical implementation plans.

Future studies should first establish stratified, longitudinal human DFU cohorts and integrate spatial multi-omics approaches to resolve macrophage and T-cell state transitions and characterize neuroimmune and stromal–endothelial communication networks. In parallel, sequencing workflows should be standardized, candidate therapeutic targets should undergo rigorous preclinical validation, and intervention strategies for recurrent DFU should be prospectively evaluated.

### 4.4 Potential of precision medicine

In precision medicine, scRNA-seq offers theoretical and experimental support for developing personalized DFU therapeutic models. Extending beyond mechanistic exploration, single-cell findings show promising value in wound classification, risk stratification, clinical decision-making and trial design (75). This technique characterizes patient-specific immune dysregulation and molecular networks of impaired angiogenesis, and identifies drug-response biomarkers to enable risk stratification, early intervention and treatment monitoring.

ScRNA-seq analyses of human wound samples advance DFU clinical translation. According to transcriptional signatures of cell subsets, DFU is categorized into inflammation-dominant, angiogenesis-deficient and matrix repair-impaired molecular subtypes, exceeding traditional classification based on wound appearance and duration, and guiding individualized strategies including anti-inflammatory therapy, pro-angiogenic treatment and matrix remodeling (78). The abundance of wound-specific endothelial cells and inflammatory macrophage subsets facilitates healing risk evaluation, enabling early detection of refractory ulcers and guiding debridement, negative pressure therapy and biological dressing administration. Targets and biomarkers identified by single-cell profiling optimize trial enrollment criteria, reduce cohort heterogeneity and enhance experimental reliability (38). Moreover, gene signatures from single-cell atlases support early identification of wound infection and prediction of ulcer recurrence, compensating for deficiencies in conventional laboratory tests. This review illustrates immune dysregulation-mediated impaired wound healing in DFU using scRNA-seq evidence, whereas several limitations persist.

At the sample level, current research relies mainly on small cross-sectional clinical specimens and lacks multi-center, large-sample longitudinal cohort validation. Fresh wound-tissue sampling is difficult, and quality control is difficult to standardize, resulting in limited data stability and generalizability (41). Numerous mechanistic discoveries are derived from diabetic mouse models, which fail to recapitulate human DFU features including neurovascular impairment, recurrent infection and comorbidities. Immune disparities between humans and mice block clinical translation. Technically, most work only uses single-cell transcriptomics without multi-dimensional validation by spatial genomics and proteomics, failing to interpret protein function and cellular spatial patterns. Current studies mainly report phenotypic correlations instead of causal evidence from *in vitro* and *in vivo* experiments. Primary pathogenic triggers cannot be easily distinguished from secondary lesions, while confounders such as age and comorbidities are underanalyzed. In the future, AI-assisted diagnosis, multi-omic biomarker panels and nanodrug delivery systems can transform DFU management from experience-based to data-driven precision therapy, enabling individualized treatment and dynamic efficacy monitoring (69).

In conclusion, single-cell omics is a milestone in reshaping DFU research toward precision medicine. Future efforts should prioritize multi-omics integration, rigorous mechanism validation, and clinical translation, including targeted sequencing of patients with recurrent ulcers. These strategies will uncover novel wound-healing mechanisms, thereby enabling early screening, tailored treatment, and precise management of chronic, non-healing wounds such as DFUs (**Figure 6**).
